# Altered Protein Profiles During Epileptogenesis in the Pilocarpine Mouse Model of Temporal Lobe Epilepsy

**DOI:** 10.3389/fneur.2021.654606

**Published:** 2021-05-28

**Authors:** Md. Mahiuddin Ahmed, Andrew J. Carrel, Yasmin Cruz Del Angel, Jessica Carlsen, Ajay X. Thomas, Marco I. González, Katheleen J. Gardiner, Amy Brooks-Kayal

**Affiliations:** ^1^Department of Neurology, University of Colorado Alzheimer's and Cognition Center, Linda Crnic Institute for Down Syndrome, University of Colorado Anschutz Medical Campus, Aurora, CO, United States; ^2^Division of Neurology and Translational Epilepsy Research Program, Department of Pediatrics, University of Colorado School of Medicine, Aurora, CO, United States; ^3^Section of Neurology and Developmental Neuroscience, Department of Pediatrics, Baylor College of Medicine, Houston, TX, United States; ^4^Section of Child Neurology, Texas Children's Hospital, Houston, TX, United States; ^5^Department of Pediatrics, Linda Crnic Institute for Down Syndrome, University of Colorado Anschutz Medical Campus, Aurora, CO, United States; ^6^Department of Pharmaceutical Sciences, Skaggs School of Pharmacy and Pharmaceutical Sciences, University of Colorado Anschutz Medical Campus, Aurora, CO, United States; ^7^Children's Hospital Colorado, Aurora, CO, United States; ^8^Department of Neurology, University of California Davis School of Medicine, Sacramento, CA, United States

**Keywords:** epileptogenesis, seizures, signaling, pilocarpine, protein phosphorylation, reverse phase protein array

## Abstract

Epilepsy is characterized by recurrent, spontaneous seizures and is a major contributor to the global burden of neurological disease. Although epilepsy can result from a variety of brain insults, in many cases the cause is unknown and, in a significant proportion of cases, seizures cannot be controlled by available treatments. Understanding the molecular alterations that underlie or are triggered by epileptogenesis would help to identify therapeutics to prevent or control progression to epilepsy. To this end, the moderate throughput technique of Reverse Phase Protein Arrays (RPPA) was used to profile changes in protein expression in a pilocarpine mouse model of acquired epilepsy. Levels of 54 proteins, comprising phosphorylation-dependent and phosphorylation-independent components of major signaling pathways and cellular complexes, were measured in hippocampus, cortex and cerebellum of mice at six time points, spanning 15 min to 2 weeks after induction of status epilepticus. Results illustrate the time dependence of levels of the commonly studied MTOR pathway component, pS6, and show, for the first time, detailed responses during epileptogenesis of multiple components of the MTOR, MAPK, JAK/STAT and apoptosis pathways, NMDA receptors, and additional cellular complexes. Also noted are time- and brain region- specific changes in correlations among levels of functionally related proteins affecting both neurons and glia. While hippocampus and cortex are primary areas studied in pilocarpine-induced epilepsy, cerebellum also shows significant time-dependent molecular responses.

## Introduction

Epilepsy affects more than 65 million people worldwide, making it the third most common neurological disorder (1). While some cases of epilepsy have a known genetic basis, others, the acquired epilepsies, arise from brain insults, such as traumatic injury, inflammation, tumor, and stroke (2). In many cases, however, the causes are unknown. Importantly, in ~30% of cases, seizures are not controlled by current therapeutic options.

Epileptogenesis is the process in which a normally functioning brain is transformed to one displaying the excessive and abnormal synchronous neuronal activity of an epileptic brain, manifested as spontaneous recurrent seizures (2, 3). Epileptogenesis is likely a continuous process developing over weeks, months, or even years beyond the initial insult (4–6). Treatments for epilepsy may therefore focus on preventing or controlling seizures after epilepsy is established, or ideally, on interrupting the damaging processes during epileptogenesis and preventing onset of spontaneous seizures all together. Understanding of the molecular processes occurring during epileptogenesis, and discriminating the deleterious from the compensatory, will help in developing novel therapeutics.

The most common focal human epilepsy is Temporal Lobe Epilepsy (TLE), representing ~60% of focal epilepsy cases. Pilocarpine injection in rodents is considered a good model of TLE because it produces characteristics observed in human epilepsy, including a seizure-free (latent) period prior to onset of recurrent seizures and development of localized lesions within the hippocampus (7, 8). Of interest also, the chronic seizures induced by pilocarpine are largely refractory to anti-seizure drugs, a situation similar to that observed in patients with TLE. The pilocarpine model and other models of acquired epilepsy such as kainate, pentylenetetrazol, and direct electrical stimulation, allow analysis of the molecular processes occurring at specific time points during epileptogenesis, from status epilepticus through the latent period to the stage of chronic spontaneous seizures. Using these models, detailed molecular observations can be made. For example, during the chronic phase after pilocarpine injection, increased levels of the protein for the immediate early gene, cFos, were seen in principal neurons within 15 min of an episode of seizures and, a few hours later, increased Fos protein was present in the cell bodies of interneurons (9). In similar studies, increased phosphorylation of ERK1/2 was seen in a subpopulation of neural progenitors specifically located within the sub-granular zone of the dentate gyrus of hippocampus (10); this disappeared within minutes of seizure onset (11). From the phenotypes caused by single gene mutations (e.g., TSC, tuberous sclerosis), a role for the Mechanistic Target of Rapamycin (MTOR) pathway has been established in epilepsy. MTOR activation, commonly assessed by levels of ribosomal protein S6 phosphorylation (pS6), has been demonstrated during epileptogenesis in rodent models (12–15). More comprehensive information on gene expression abnormalities can be obtained from whole transcriptome studies via RNA microarrays. Hansen et al. found that pilocarpine caused transcriptional changes influencing the MAPK pathway (16). Integrating results from multiple such SE rodent model transcriptome studies, Chen et al. identified common differentially expressed genes; “hub” genes were enriched in those related to inflammatory responses and microglial/macrophage activation (17). A limitation of these latter studies remains that RNA levels do not robustly predict protein levels, and perturbations in levels of protein modifications/activations, critical to driving cellular changes, necessarily remain unknown. Mass spectrometry-based large-scale proteomic studies have also been carried out in rodent models (18–21). Such studies have identified additional candidate proteins and pathways but, notably, failed to identify commonly studied components of the MTOR and MAPK pathways. Thus, knowledge of critical perturbations occurring in rodent models of TLE, and by extension human TLE, remain to be robustly established.

To this end, we have used the moderate throughput technique of reverse phase protein arrays (RPPA). We measured levels of 54 components of major signaling pathways and cellular processes, in hippocampus, cortex and cerebellum at six time points after injection with pilocarpine and induction of status epilepticus. The results include demonstration of complex sequential alterations in levels of components of the MTOR and MAPK pathways and in subunits of the N-methyl-D-aspartate receptor (NMDAR) receptor. They also show correlations among levels of pathway and receptor components in saline treated mice, and how these correlations are lost and altered over time with pilocarpine. Components of the JAK/STAT and apoptosis pathways also show significant time-dependent alterations. While hippocampus, the brain region most affected during pilocarpine-induced epileptogenesis, shows large numbers of perturbations, cortex and cerebellum also display significant and divergent responses in pathways that are critical for brain function. Together, these data present a novel picture of the complexity of protein responses during epileptogenesis. These may provide targets for novel therapeutics aimed at disrupting the epileptogenic processes and preventing development of spontaneous seizures.

## Materials and Methods

All animal procedures were performed in accordance with the regulations of the institutional animal care and use committees of the University of Colorado Anschutz Medical Campus and the National Institutes of Health *Guide for the Care and Use of Laboratory Animals*.

### Pilocarpine-Induced Status Epilepticus

A total of 160FVB/NJ mice (all males JAX stock #001800) were purchased from The Jackson Laboratory (Bar Harbor, ME) and received at 5–7 weeks of age. Prior to experiments, littermates (on average, 5) were group housed in temperature- and humidity-controlled rooms on a 12 h light/dark cycle with access to food and water *ad libitum*. All mice were allowed to acclimate to the environment and altitude for 1 week prior to handling. Prior to the start of experiments, mice (18–24 g) were briefly handled once daily for at least 1 week to reduce the stress induced by experimental protocols. On the day of the injections, mice were transferred to an experimental room, marked, weighed, and allowed to habituate undisturbed for at least 1 h. Entire litters (an average of 5 mice) were randomly assigned to a single treatment/time point group. Only in cases where fewer than 5 mice comprised a single litter were mice from two litters combined for a single treatment/time point. For pilocarpine treated mice, 15 min before the first pilocarpine injection, mice were given an intraperitoneal injection of 1 mg/kg scopolamine methyl bromide (Sigma-Aldrich) to block the peripheral muscarinic effects of pilocarpine. The initial dose of pilocarpine HCl (200 mg/kg; Sigma-Aldrich) was followed, after 1 h, by subsequent doses (100 mg/kg) given at 30 min intervals until the onset of SE, defined as the appearance of repeated behavioral seizures (stage four or higher with at least one seizure being five or higher) according to a modified Racine scale (22, 23). In the majority animals, SE initiated within 1 h of the first pilocarpine injection; thus these animals received a single injection. The maximum number of injections given was three. SE persisted for at least 90 min. Control mice were given injections of saline at identical time intervals. After SE induction, mice were returned to their home room, singly housed, and given free access to water and moistened chow. Cohorts (5 per group) of pilocarpine treated mice were sacrificed after SE, at 15 min (onset), 1 and 6 h, 1 and 5 days, and 2 weeks. Groups of saline-injected mice were sacrificed at the same time points (see Supplementary Figure 1).

### Tissue Processing and Preparation of Protein Lysates

In order to preserve the endogenous levels of protein phosphorylation, mice were sacrificed by cervical dislocation without anesthetic. The whole brain was rapidly removed, immediately snap frozen in liquid nitrogen, and stored at −80°C. Before lysate preparation, the brains were removed from the freezer and, without thawing, rapidly heated to 95°C under vacuum conditions using the Stabilizor T1 (Denator, AB), as described previously (24). The cortex, hippocampus, and cerebellum were dissected out, weighed, placed in 10 volumes of IsoElectric Focusing (IEF) buffer (8 M urea, 4% CHAPS, 50 mM Tris) and homogenized by sonication with three bursts of 5 s in a Branson Sonic Power Co. (Danbury, CT). Homogenates were centrifuged at 14,000 rpm for 30 min at 4°C to remove debris, and the protein concentration of the cleared supernatant was determined using the 660 nM Protein Assay Kit (Pierce); all sample protein concentrations were within the range of 9–11 mg/ml.

### Antibodies and Validation for RPPA

Reverse phase protein arrays (RPPA) require highly specific antibodies. Prior to use, the specificity of each lot of each antibody was verified by Western blot using mouse brain lysates to show that only clean band(s) of explainable size, with no non-specific bands, were present. All secondary antibodies (IgG; anti-goat, rabbit, and mouse) have been shown previously to produce signals that are <5% above local background when incubated with an RPPA slide in the absence of any primary antibody; signals of these levels are too low to be reliably quantitated and were ignored in data analysis. Proteins screened for expression level are listed in Supplementary Table 1, with antibody information regarding source, catalog number, and dilution factor.

### Array Assembly, Printing, and Staining

Each sample lysate was prepared in three replicates of a 5-point serial dilution, with a 0.8 dilution factor, and 1 buffer control, in a 384-well V-shaped AB gene plate (Thermo Fisher Scientific, Rockford, IL). Samples were printed, in triplicate, onto nitrocellulose-coated glass slides (Grace Bio-Laboratories, Inc., Bend, OR) using an Aushon BioSystems 2470 Arrayer (Aushon BioSystems, Billerica, MA) with 185-μm pins and a single touch. The arrays were produced in two major print runs and slides were stored at 4°C until further use. For protein detection, slides were incubated in blocking solution (3% BSA, Sigma, USA) in TBST (Tris-buffered saline, 0.1% Tween 20) for 4 h, followed by overnight incubation at 4°C with shaking with the primary antibody (antibody dilutions are provided in Supplementary Table 1). Detection of the bound primary antibody was performed by incubation with the secondary antibody, Fluorescence Alexa Fluor 555 goat anti-mouse or anti-rabbit or rabbit anti-goat (1:2,000 dilution) (Invitrogen, Carlsbad, CA), for 90 min at room temperature. Slides were washed and dried, and signals were detected by scanning on a PerkinElmer Scan Array Express HT Microarray Scanner (PerkinElmer Inc., MA, USA). For normalization, total protein for each spot was determined by staining three non-sequential slides from each print run with SyproRuby reagent (Invitrogen, CA, USA) following the manufacturer's protocol.

### Image Analysis, Quantification, Normalization, and Statistical Analysis

Signals on each slide were quantified using Scan Array Express software (PerkinElmer Inc., MA, USA). After removal of technical outliers, each SyproRuby-normalized protein value was included in the statistical analyses if the level was within its mean ±3 standard deviations. Mean differences between groups were reported as a percent change, assessed using a hierarchical three-level mixed effects model to account for possible correlations and variability between replicates and dilutions within each sample. The Benjamini-Hochberg corrected *p* < 0.05 with a false discovery rate (FDR) of 5% was considered for overall statistical significance across the entirety of the hypotheses. Supplementary Tables 2–4 contain results (% differences, *p*-value. and FDR) for all measured proteins in the hippocampus, cortex, and cerebellum for, respectively, the pilocarpine vs. saline, saline vs. saline chronic, and pilocarpine vs. saline-chronic comparisons. To assess possible relationships between levels of all pairs of proteins, Spearman correlation analysis was carried out. Protein values were reduced to one observation per mouse for each brain region of each individual of each treatment group and used to compute Spearman correlation coefficients. Graphs for data from protein pairs with correlation coefficients >0.8 with *P* < 0.05 were manually inspected and those with non-linear relationships were eliminated; the remainder were used to generate correlation network figures. All data analyses were carried out using SAS® version 9.3 (SAS Institute Inc., Cary, NC). Additional details of quantification and review of data quality and reproducibility were carried out as described previously (25–27).

## Results

The goal of these experiments was to determine the time courses of protein expression changes affecting important signaling pathways in a pilocarpine model of acquired epilepsy. Levels of 54 proteins were measured in hippocampus, cortex and cerebellum, at six time points after pilocarpine injection: at ~15 min (after onset of seizure activity), at 1, 6, and 24 h (acute period), at 5 days (the latent period, with no spontaneous seizure activity), and at 2 weeks (during the chronic period, characterized by spontaneous recurrent seizures). Saline-injected mice served as controls and cohorts were sacrificed at the same time points post-injection as pilocarpine treated mice. Proteins measured included 18 components of the classical MAP kinase pathway (10 to phosphorylation-specific forms and 8 to phosphorylation-independent forms), 16 components of the MTOR pathway (6 to phosphorylation-specific forms), and 8 subunits of ionotropic glutamate receptors (5 for NMDA receptors, including 3 phosphorylation-specific forms, and 3 for AMPA receptor subunits, including one phosphorylation-specific form). Additional proteins included components of apoptosis and JAK/STAT pathways. Not all proteins of interest could be measured because of the requirement for highly specific antibodies, free of significant background signals. A complete list of the proteins analyzed is provided in Supplementary Table 1.

Given two treatment groups and six time points, many protein expression comparisons can be made. Three were selected as the most biologically informative. The first, pilocarpine/saline, is the direct comparison of pilocarpine treated to saline treated mice at corresponding time points post-injection. The second, saline/saline-chronic, is the comparison of saline treated mice at each time point to saline treated mice at the chronic time point. No expression changes would be expected to be induced by saline, beyond responses to the stress of handling and injection experience, and these would be expected to dissipate early in the time course. However, saline treated mice were housed in the same room as pilocarpine injected mice and thus were exposed to the stress of seizure experience in their littermates. This “observer” status (or other unknown environmental influences) indeed turned out to be associated with significant protein expression changes throughout the time course. To describe these changes, for the second comparison, we assumed that the saline-chronic expression pattern most closely approximated the expression pattern of untreated mice, because by this time, mice have had 3 weeks to adjust to the post-injection environment. The third comparison, pilocarpine/saline-chronic, describes protein levels in pilocarpine mice at each of the 6 time points relative to those in saline treated mice from the chronic time point. This was chosen to best describe pilocarpine-induced differences in expression from an approximately normal baseline. Data for all proteins in the three brain regions and three comparisons are provided in Supplementary Tables 2–4.

### Pilocarpine/Saline Comparison

**Figure 1:**
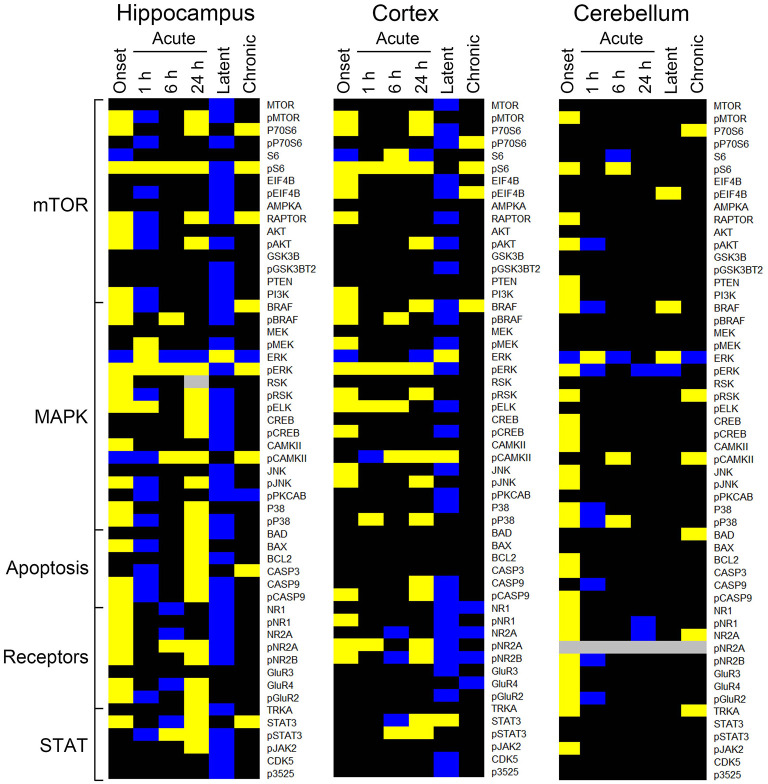
Changes in protein expression during epileptogenesis. Heatmaps show proteins with an average expression level in pilocarpine treated mice significantly different from saline treated mice at the corresponding time points. Only proteins with differences ≥15% as determined by a three level mixed effects model, with *p* ≤ 0.0005, and a Benjamini-Hochberg correction for multiple testing applied with a false discovery rate of 0.05 are shown. Data derived from Supplementary Table 2. Proteins are grouped by general category. Yellow, increased; blue, decreased; black, no difference; gray, not measured. *n* = 5 for each group, except for chronic pilocarpine where *n* = 4.

The heatmaps in Figure 1 compares protein expression, in the three brain regions, between pilocarpine and saline treated mice at corresponding time points. Table 1 lists the number of proteins whose levels are increased and decreased in each brain region at each time point. In hippocampus (Table 1 and Figure 1), at onset of seizures, there are significant elevations in the levels of 28 proteins, among them, multiple components of the MTOR and MAPK pathways. These include not only the commonly measured pS6 and pERK, but also pMTOR, RAPTOR, AKT, and pAKT, as well as kinases of the MAPK pathway pBRAF and pRSK, and the downstream transcription factor, pELK. Multiple subunits of NMDA and AMPA receptors are also elevated at onset. These initial increases are followed at 1 h by decreases in expression of a total of 20 proteins, 12 of which were initially elevated. This is a bimodal pattern with a second round of increases (in 28 proteins) present at 24 h, followed again by decreases (in 40 proteins) during the latent period. Lastly, during the chronic phase, 8 proteins remain elevated and 2 are repressed compared with saline mice. Among these are pS6 and RAPTOR from the MTOR pathway and BRAF and pERK from the MAPK pathway.

**Table 1:** Summary of protein expression changes.

**Brain region**	**Onset**	**1 h**	**6 h**	**24 h**	**Latent**	**Chronic**
**Pilocarpine/saline**						
HP	28,3 (17)	5,20 (7)	7,5	28,1 (18)	1,40 (23)	8,2
CR	20,2 (4)	5,1	8,3	17,2 (8)	3,29 (11)	4,4
CB	30,1 (13)	1,5	3,2	0,3	3,1	6,1
**Saline/saline-chronic**						
HP	0,6	5,2	10,0	1,21	9,1	na
CR	0,24 (2)	0,5	1,11	1,16	5,2	na
CB	8,6	18,1	13,1	10,2	18,1	na
**Pilocarpine/saline-chronic**						
HP	14,1	3,11	14,1	8,6	1,33 (14)	2,8
CR	2,3	5,9	7,5	3,4	1,25 (7)	4,4
CB	30,1 (10)	13,2	11,1	1,1	24,1 (5)	6,1
**Pilocarpine/saline-latent**						
HP	6,2	3,25 (10)	7,2	3,7	1,49 (23)	1,3
CR	2,6	7,13	5,0	6,6	3,29 (11)	3,5
CB	6,0	5,8	0,1	2,5	3,1	0,2

*Number of proteins with significantly increased or decreased levels respectively in each treatment group, per time point, and brain region. Numbers in brackets indicate the numbers of proteins with magnitudes of change ≥25%*.

Similar to hippocampus, cortex and cerebellum also showed increased levels of many proteins at onset (20 and 30, respectively; Table 1 and Figure 1), and several were common to those in hippocampus, notably pS6 and pERK, and pMTOR, RAPTOR, BRAF, pRSK, pCASP9, pJNK, pNR1, 2A, and 2B. Cortex does not, however, show the large number of decreased protein levels at 1 h, and has fewer increased proteins at 24 h (only 17 vs. 28 in hippocampus). Cortex does however show a large number (28) of decreases in protein levels during the latent period. Cerebellum was unique in rapidly stabilizing protein expression, showing few changes (only 3–7 in total) at each time point after onset.

Tables 2–4 provide the magnitudes of protein expression differences for components of the MAPK and MTOR pathways, plus apoptosis, and JAK/STAT related proteins in hippocampus, cortex and cerebellum. Of note, in the MAPK pathway, although BRAF, MEK, ERK, and RSK are sequential in the kinase cascade, differences in phosphorylation levels (from saline injected mice) are not necessarily correlated in direction and magnitude. For example, in hippocampus at onset, while pBRAF and pERK levels have increased, the level of the intermediate pMEK has decreased. In cortex at onset, pBRAF and pMEK each increased by ~25%, but pERK increased by 170%. In general, in both brain regions, while the direction of change is consistent, the magnitude of pERK change tends to be greater than those for pBRAF and pMEK. The ratios of phosphorylated form to total kinase, indicating the ratio of activated kinase, also change with time in a non-uniform fashion. For example, in hippocampus at 24 h, the total ERK level has decreased by 28% while the level of pERK has increased 71%, indicating a 2.4-fold increase in the proportion of activated ERK; in contrast, during the latent period, proportions of pERK/ERK are decreased by 0.4-fold from those in saline mice. Alternatively, the level of RSK protein is stable after the initial increase at onset, while levels of pRSK range from −32 to +37%. Cortex also shows disproportionate changes in activation; ratios of pBRAF/BRAF do not change as dramatically throughout the time course, while those of pERK/ERK approximately double at onset and 6 h. Large variations during the sub-acute period are seen in levels of pCAMKII, in hippocampus changing from −45% at 1 h to +83% at 24 h, and in cortex, changing from −29 to +102%. Cerebellum does not show great differences in sequential kinase phosphorylation levels, nor time changes in activation ratios. One notable feature common to all three brain regions are the magnitudes of responses of phosphorylated P38. In both hippocampus and cerebellum, pP38 increases by 40% at onset and in cortex, by 47% later, at 24 h.

**Table 2:** Protein changes in hippocampus, MAPK, MTOR, JAK/STAT, and apoptosis pathway components (pilocarpine vs. saline at corresponding time points).

**Protein**	**Onset**	**1 h**	**6 h**	**24 h**	**Latent**	**Chronic**
**MAPK**						
BRAF	19%	−23%	–	11%	−25%	19%
pBRAF	26%	–	22%	12%	−15%	–
MEK	–	–	–	–	−14%	–
pMEK	−11%	15%	–	11%	−30%	–
ERK	−37%	39%	−15%	−28%	21%	−25%
pERK	24%	–	13%	71%	−43%	22%
RSK	42%	–	–	–	–	–
pRSK	30%	−16%	−12%	37%	−32%	–
pELK	28%	17%	–	37%	−25%	–
CREB	12%	−10%	–	21%	−26%	–
pCREB	10%	−12%	–	23%	−19%	–
CAMKII	17%	11%	–	9%	−28%	–
pCAMKII	−22%	−45%	21%	83%	12%	38%
JNK	12%	−13%	–	10%	−27%	–
pJNK	34%	−16%	13%	37%	−22%	–
P38	20%	–	–	30%	–	–
pP38	40%	−16%	–	9%	−24%	–
**MTOR**						
MTOR	–	−14%	–	–	−17%	–
pMTOR	27%	−24%	–	24%	−30%	–
P70S6	18%	−14%	–	46%	−14%	28%
pP70S6	12%	−26%	–	–	−31%	–
S6	−17%	–	–	–	−14%	–
pS6	33%	26%	133%	73%	−32%	44%
EIF4B	11	−13%	–	13%	−24%	–
pEIF4B	12%	−16%	15%	−11%	−26%	–
AMPKA	–	–	–	–	−10%	–
RAPTOR	28%	−19%	–	17%	−35%	20%
AKT	21%	−16%	–	13%	−27%	–
pAKT	23%	−20%	–	26%	−36%	11%
PTEN	8%	10%	–	–	−21%	–
PI3K	21%	−15%	–	–	−17%	–
**APOPTOSIS, JAK/STAT**						
BAD	14%	−12%	–	16%	−22%	–
BAX	20%	−17%	–	17%	−10%	–
BCL2	12%	−11%	–	21%	−30%	–
CASP3	13%	−18%	–	16%	−13%	21%
CASP9	21%	−19%	14%	23%	−34%	–
pCASP9	31%	−26%	–	28%	−27%	–
STAT3	25%	−14%	−16%	68%	–	55%
pSTAT3	–	−15%	32%	30%	−18%	–
pJAK2	–	–	–	18%	−17%	–
TRKA	13%	–	−12%	25%	−18%	–

*Data extracted from Supplementary Table 2. All values are significant as determined by a three-level mixed effects model, with p ≤ 0.005 and the Benjamini-Hochberg correction for multiple testing, with false discovery rate (FDR) p ≤ 0.05. Only significant differences ≥|10%| are shown*.

**Table 3:** Protein changes in cortex, MAPK, MTOR, JAK/STAT, and apoptosis pathway components (pilocarpine vs. saline at corresponding time points).

**Protein**	**Onset**	**1 h**	**6 h**	**24 h**	**Latent**	**Chronic**
**MAPK**						
BRAF	17%	–	–	19%	−25%	17%
pBRAF	28%	–	32%	13%	−19%	–
MEK	–	–	–	–	−15%	–
pMEK	24%	−13%	15%	–	−20%	–
ERK	−20%	–	–	−42%	49%	–
pERK	170%	36%	95%	28%	−37%	–
RSK	42%	–	–	–	–	–
pRSK	23%	–	–	22%	–	–
pELK	18%	38%	18%	14%	−19%	−10%
CREB	–	–	–	–	−11%	–
pCREB	17%	–	–	12%	−17%	–
CAMKII	11%	–	–	10%	−12%	–
pCAMKII	–	−29%	62%	102%	26%	–
JNK	23%	–	–	13%	−19%	–
pJNK	22%	–	–	33%	–	–
P38	10%	–	–	–	–	–
pP38	–	29%	–	47%	−14%	–
**MTOR**						
MTOR	13%	–	–	–	17%	–
pMTOR	28%	–	–	23%	−12%	–
P70S6	17%	–	–	42%	−21%	–
pP70S6	–	–	–	–	−25%	19%
S6	−17%	–	15%	−23%	–	−13%
pS6	32%	87%	175%	67%	–	27%
EIF4B	25%	–	–	14%	−23%	–
pEIF4B	20%	–	–	–	−25	34%
AMPKA	–	–	–	–	–	–
RAPTOR	19%	–	–	15%	−19%	12%
AKT	–	–	–	–	−11%	–
pAKT	11%	–	–	16%	−18%	–
PTEN	–	–	–	–	−11%	–
PI3K	23%	−10%	–	13%	−14%	–
**APOPTOSIS, JAK/STAT**						
BAD	14%	–	–	–	−13%	–
BAX	–	–	–	11%	−10%	–
BCL2	–	–	–	–	−14%	–
CASP3	–	–	–	10%	−11%	–
CASP9	–	–	–	24%	−25%	–
pCASP9	24%	–	–	24%	−18%	–
STAT3	10%	–	−23%	63%	22%	–
pSTAT3	–	–	22%	18%	–	12%
pJAK2	–	–	–	–	–	–
TRKA	–	–	–	–	−12%	–

*Data extracted from Supplementary Table 2. All values are significant as determined by a three-level mixed effects model, with p ≤ 0.005 and the Benjamini-Hochberg correction for multiple testing, with false discovery rate (FDR) p ≤ 0.05. Only significant differences ≥|10%| are shown*.

**Table 4:** Protein changes in cerebellum, MAPK, MTOR, JAK/STAT, and apoptosis pathway components (pilocarpine vs. saline at corresponding time points).

**Protein**	**Onset**	**1 h**	**6 h**	**24 h**	**Latent**	**Chronic**
**MAPK**						
BRAF	21%	−21%	–	–	16%	–
pBRAF	10%	–	–	–	–	11%
MEK	10%	–	−10%	–	–	–
pMEK	13%	–	–	–	–	–
ERK	−30%	28%	−22%	–	16%	−31%
pERK	24%	−23%	–	17%	−16%	–
RSK	–	−14%	–	–	–	–
pRSK	31%	−12%	–	–	−10%	21%
pELK	–	–	–	–	–	–
CREB	29%	–	–	–	–	13%
pCREB	25%	–	–	–	–	14%
CAMKII	25%	–	–	–	–	13%
pCAMKII	–	–	23%	–	–	30%
JNK	17%	–	–	–	–	–
pJNK	32%	−12%	–	–	–	13%
P38	34%	–	–	–	–	–
pP38	40%	–	22%	–	–	–
**MTOR**						
MTOR	–	–	–	–	–	–
pMTOR	25%	–	–	–	–	–
P70S6	–	–	–	–	–	19%
pP70S6	–	–	–	–	9%	12%
S6	–	–	−19%	–	–	–
pS6	24%	–	23%	–	–	–
EIF4B	15%	–	–	–	–	–
pEIF4B	–	–	10%	−14%	27%	–
AMPKA	10%	–	–	–	–	10%
RAPTOR	19%	–	12%	–	–	–
AKT		–	–	–	–	–
pAKT	18%	−18%	–	–	–	–
PTEN	18%	–	–	–	–	–
PI3K	17%	–	–	–	–	–
**APOPTOSIS, JAK/STAT**						
BAD	15%	–	–	–	–	19%
BAX	13%	–	–	–	–	−11%
BCL2	18%	–	–	–	–	–
CASP3	23%	−11%	–	–	–	14%
CASP9	–	−20%	–	–	–	–
pCASP9	23%	–	–	–	10%	11%
STAT3	14%	–	–	–	–	–
pSTAT3	–	14%	–	14%	–	11%
pJAK2	17%	–	–	–	–	–
TRKA	16%	−10%	–	–	–	19%

*Data extracted from Supplementary Table 2. All values are significant as determined by a three-level mixed effects model, with p ≤ 0.005 and the Benjamini-Hochberg correction for multiple testing, with false discovery rate (FDR) p ≤ 0.05. Only significant differences ≥|10%| are shown*.

By the chronic stage, only 4 and 3 of the 17 MAPK components remain abnormal in hippocampus and cortex, respectively, suggesting that, while many proteins are responsive in the early stages post-SE, abnormalities in these proteins largely are not playing roles in the production of chronic seizures. This differs in cerebellum, where 9 MAPK components remain abnormal, in particular pRSK and pCAMKII levels are elevated by 21 and 30%, respectively, and ERK is decreased by 31%.

Observations of the MTOR pathway are qualitatively similar to those of MAPK. In both hippocampus and cortex, levels of total MTOR are largely stable, while levels of pMTOR range from −30 to +27% in hippocampus and −12 to +28% in cortex. While levels of pS6 are constantly elevated in the sub-acute stages, with the largest elevation of any protein, 133 and 175% seen in hippocampus and cortex, respectively, at 24 h, neither levels of S6 nor the precursor kinase, pP70S6, change dramatically. It is of interest that levels of pMTOR, RAPTOR, and pAKT show the similar magnitudes of increases and decreases through the sub-acute and latent stages in both regions. In cerebellum, there are fewer and smaller changes in MTOR: pS6 is elevated by ~25% at onset and 6 h, while pEIF4B is uniquely elevated during the latent period. During the chronic phase, pS6 remains elevated in cortex and hippocampus, and pEIF4B uniquely elevated in cortex.

Lastly, Tables 2–4 include proteins involved in apoptosis and JAK/STAT signaling. In hippocampus, the pro-apoptotic proteins, BAD, and BAX, show similar patterns and magnitudes of changes as the anti-apoptotic protein, BCL2 throughout the sub-acute time points. In both hippocampus and cortex, all three proteins are decreased at the latent stage. Cerebellum is unique in abnormal levels in the chronic stage, elevated BAD and BCL2, and decreased BAX. Hippocampus shows the most changes in both CASP9 and pCASP9, with the latter varying ±25 to 30% from onset through the latent stage. Similar to hippocampus, cortex has elevated levels of CASP9 and pCASP9 at 24 h, but repressed levels during the latent period. CASP3 variations have similar patterns to CASP9, but more modest magnitude of change, and remains elevated during the chronic stage in both hippocampus and cerebellum. STAT3 and pSTAT3 both change throughout the entire time course in hippocampus, with pSTAT3 most strongly elevated at 24 h and in the latent period. STAT3 is elevated by 68% at 24 h; it is elevated again, at 55%, during the chronic stage, the largest perturbation at this stage of all proteins and brain regions. Cortex also shows a strong elevation of STAT3, 63% at 24 h.

**Figure 2:**
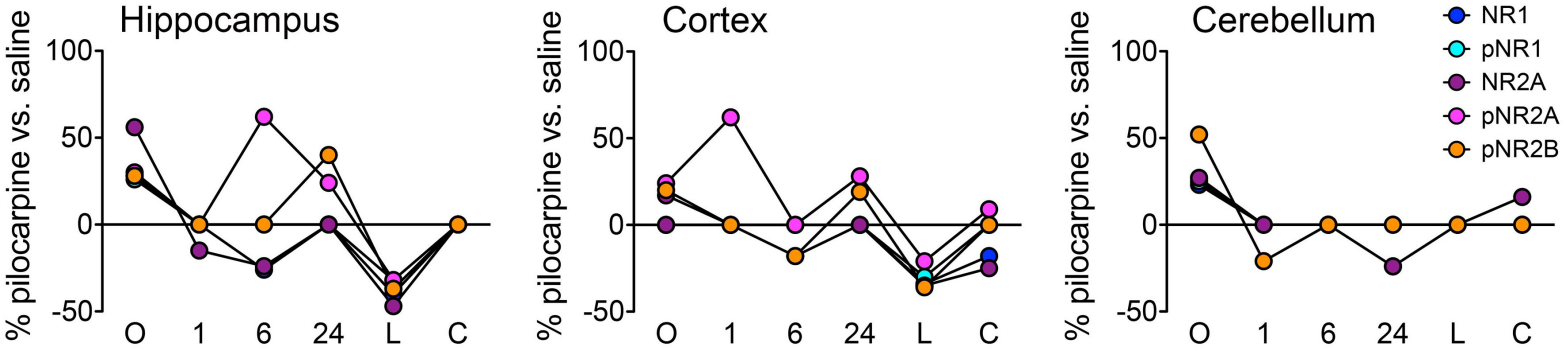
Time course of expression changes for NMDA receptor subunits. Percent changes in protein levels in pilocarpine vs. saline mice at the corresponding time points were obtained using a three-level mixed effects model; data are from Supplementary Table 2. *n* = 5 for each group except for chronic pilocarpine where *n* = 4.

Figure 2 illustrates the details of expression changes for the NMDA receptor subunits. In hippocampus, at onset, all 5 subunits are elevated, most at ~25–30%, but NR2A at >55%. By 6 h, levels of 4 are decreased by ~25% or are not different from those in saline mice. However, now pNR2A is elevated by >60%. During the latent period, all subunits are strongly repressed, but return to control levels during the chronic period. In cortex, only pNR2A shows strong variability, with maxima at 1 and 24 h. Levels of other subunits tend to decrease from highs at onset to levels repressed by 20–40% at the latent time point. Cerebellum shows a much simpler profile; after initial elevated levels, most subunits show few differences from saline controls; only pNR2A and pNR2B are decreased by ~25% at 1 and 24 h, respectively.

Together these data emphasize that hippocampus is the most affected by pilocarpine and that cortex is also similarly, if less dramatically perturbed. However, the data also show that cerebellum nevertheless displays significant responses in critical signaling pathways, particularly at onset, and that notably, of these proteins, cerebellum displays the largest number of abnormalities chronically.

### Saline/Saline-Chronic Comparison

**Figure 3:**
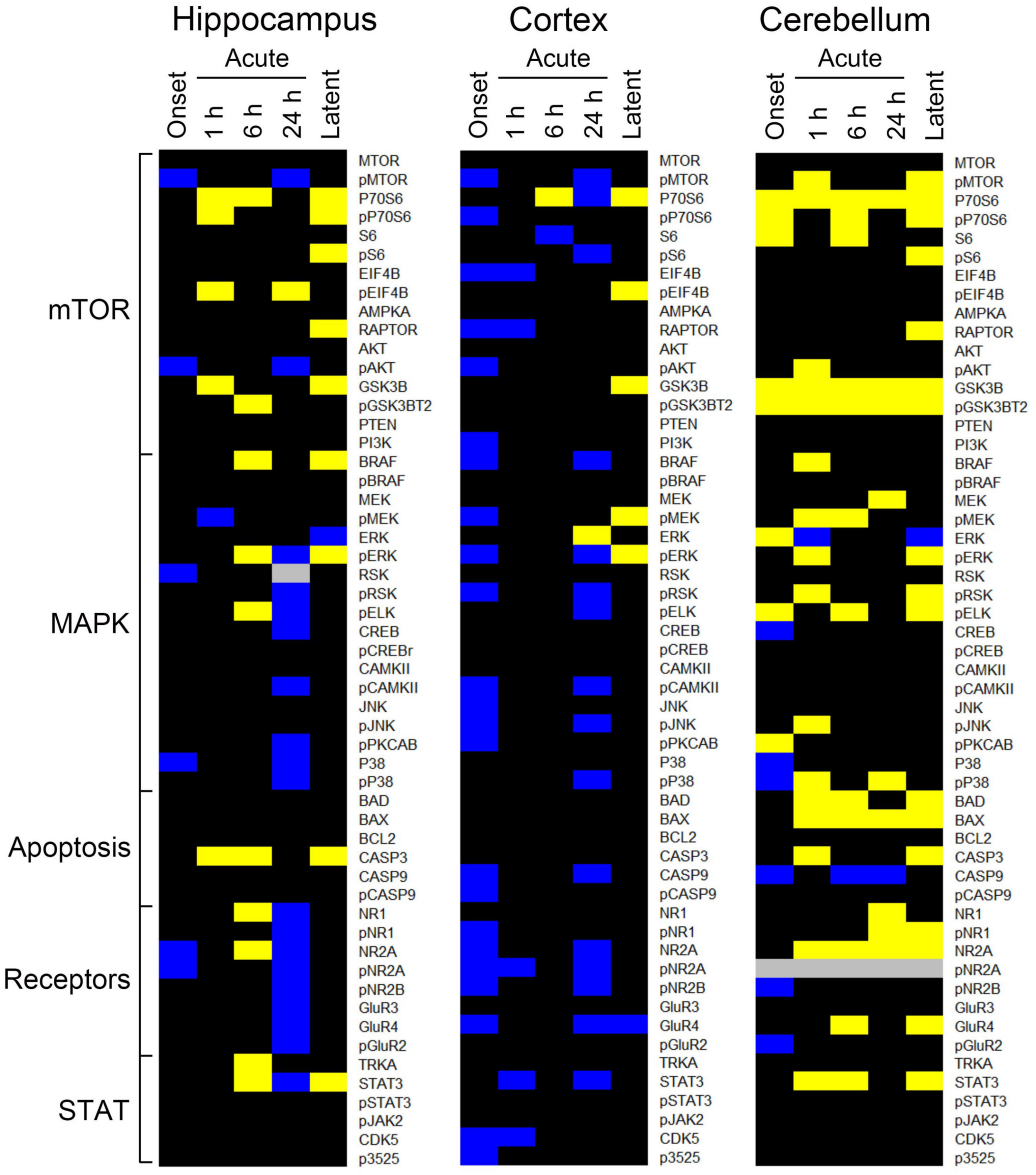
Changes in protein expression in saline-treated mice. Heatmaps show proteins with an average expression level in saline treated mice at the first five time points significantly different from saline treated mice at the chronic time point. Only proteins with differences ≥15% as determined by a three level mixed effects model, with *p* ≤ 0.0005, and a Benjamini-Hochberg correction for multiple testing applied with a false discovery rate of 0.05 are shown. Data derived from Supplementary Table 3. Proteins are grouped by general category. Yellow, increased; blue, decreased; black, no difference; gray, not measured. *n* = 5 for each group.

Figure 3 shows heatmaps for protein expression at five time points in saline treated mice compared to the saline chronic time point. Each brain region shows many alterations. In hippocampus (Figure 3), there are a large number of decreases (20) at 24 h, among them all measured subunits of NMDA and AMPA receptors, pERK and additional MAPK pathway components. Notably, there are few alterations in the MTOR pathway. In cortex, the majority of decreases occur at onset and 24 h, 24 and 16, respectively. Of the latter, 11 are also repressed in hippocampus, including pNR2A and pNR2B, pMTOR and several MAPK components. In contrast, in cerebellum, the majority of changes are increases, e.g., 18, 10, and 18 are elevated at 1 and 24 h, and during the latent period, respectively. P70S6, GSK3B, and pGSK3B are among those proteins elevated at all time points, and NR2A and BAX are elevated at all time points after onset. Together, these data indicate that the experience of saline treated mice is not totally benign, and that it develops with time. The pattern of changes may be due solely to the injection of saline; however, observing, in close proximity, the trauma of pilocarpine treated littermates may induce stress and be responsible for some responses.

### Pilocarpine/Saline-Chronic Comparison in Cerebellum

Because protein expression in saline injected mice is dynamic, it may be more realistic to compare pilocarpine data at each time point to data from the saline chronic time point, with the assumption that this latter time point most closely resembles expression of untreated mice. We examined data from cerebellum in detail because this brain region showed the greatest number and magnitude of changes in the saline chronic comparison (Figure 3 and Supplementary Table 3). Table 5 shows the pilocarpine-induced changes in cerebellum when compared with protein levels in the saline-chronic stage. At onset, MAPK pathway responses are not dramatically different from the previous comparison with the exception of P38 and pP38, where both are decreased from 34% and 40 to 12% and 18%. During the latent period, however, now 10 of 17 MAPK components are altered, where previously only 4 were. More differences are seen in the MTOR pathway, where at onset, MTOR, P70S6, S6, and AKT, that were unaffected, are now elevated between 19–41%, and pS6 and AMPKA are increased to 39 and 19%, respectively, from 24 to 10%. In addition, P70S6, pP70S6, and pS6 that were largely unresponsive throughout the time frame, now remain elevated at most sub-acute times and during the latent period. Indeed, 12 of 14 MTOR pathway proteins are now altered during the latent period, where previously only a single protein, pEIF4B, was. The apoptosis proteins, BAD and BAX are now elevated to ~25% from ~14%, and also now remain increased throughout the entire time course. Similarly, STAT3 and pSTAT3 are also increased at more time points and to higher levels. Overall, cerebellum shows more abnormalities during the latent period and appears more similar to hippocampus and cortex.

**Table 5:** Protein changes in cerebellum, MAPK, MTOR, JAK/STAT, and apoptosis pathway components (pilocarpine vs. saline chronic).

**Protein**	**Onset**	**1 h**	**6 h**	**24 h**	**Latent**	**Chronic**
**MAPK**						
BRAF	–	–	–	–	15%	–
pBRAF	22%	12%	–	–	10%	11%
MEK	16%	–	−10%	–	12%	–
pMEK	23%	19%	–	12%	–	–
ERK	−15%	–	−29%	–	−16%	−31%
pERK	19%	–	–	−27%	–	–
RSK	14%	–	–	–	–	–
pRSK	24%	–	12%	–	–	20%
pELK	29%	–	–	–	18%	–
CREB	–	−10%	–	–	–	–
pCREB	13%	–	–	–	13%	–
CAMKII	14%	–	–	–	11%	–
pCAMKII	–	–	–	–	–	30%
JNK	–	–	–	–	14%	–
pJNK	23%	–	18%	–	18%	–
P38	12%	−14%	–	–	–	–
pP38	18%	–	29%	–	23%	–
**MTOR**						
MTOR	19%	13%	–	–	17%	–
pMTOR	14%	–	–	–	19%	–
P70S6	41%	40%	31%	–	20%	19%
pP70S6	–	27%	25%	14%	26%	12%
S6	22%	–	–	27%	–	–
pS6	39%	38%	24%	–	37%	–
EIF4B	–	–	–	–	−11%	–
pEIF4B	–	25%	–	–	28%	–
AMPKA	24%	18%	–	–	11%	10%
RAPTOR	–	–	14%	–	23%	–
AKT	19%	12%	–	–	11%	–
pAKT	13%	–	15%	–	23%	–
PTEN	15%	–	–	–	–	–
PI3K	11%	–	–	–	14%	–
**APOPTOSIS, JAK/STAT**						
BAD	26%	17%	15%	11%	23%	19%
BAX	25%	18%	15%	12%	19%	11%
BCL2	17%	–	–	–	17%	–
CASP3	18%	–	13%	–	21%	14%
CASP9	–	−21%	–	–	–	–
pCASP9	22%	–	–	–	14%	11%
STAT3	24%	–	–	–	18%	–
pSTAT3	15%	22%	–	12%	–	11%
pJAK2	–	–	–	–	–	–
TRKA	–	–	–	–	–	19%

*Data extracted from Supplementary Table 4. All values are significant as determined by a three-level mixed effects model, with p ≤ 0.005 and the Benjamini-Hochberg correction for multiple testing, with false discovery rate (FDR) p ≤ 0.05. Only significant differences ≥|10%| are shown*.

### Correlation Networks

**Figure 4:**
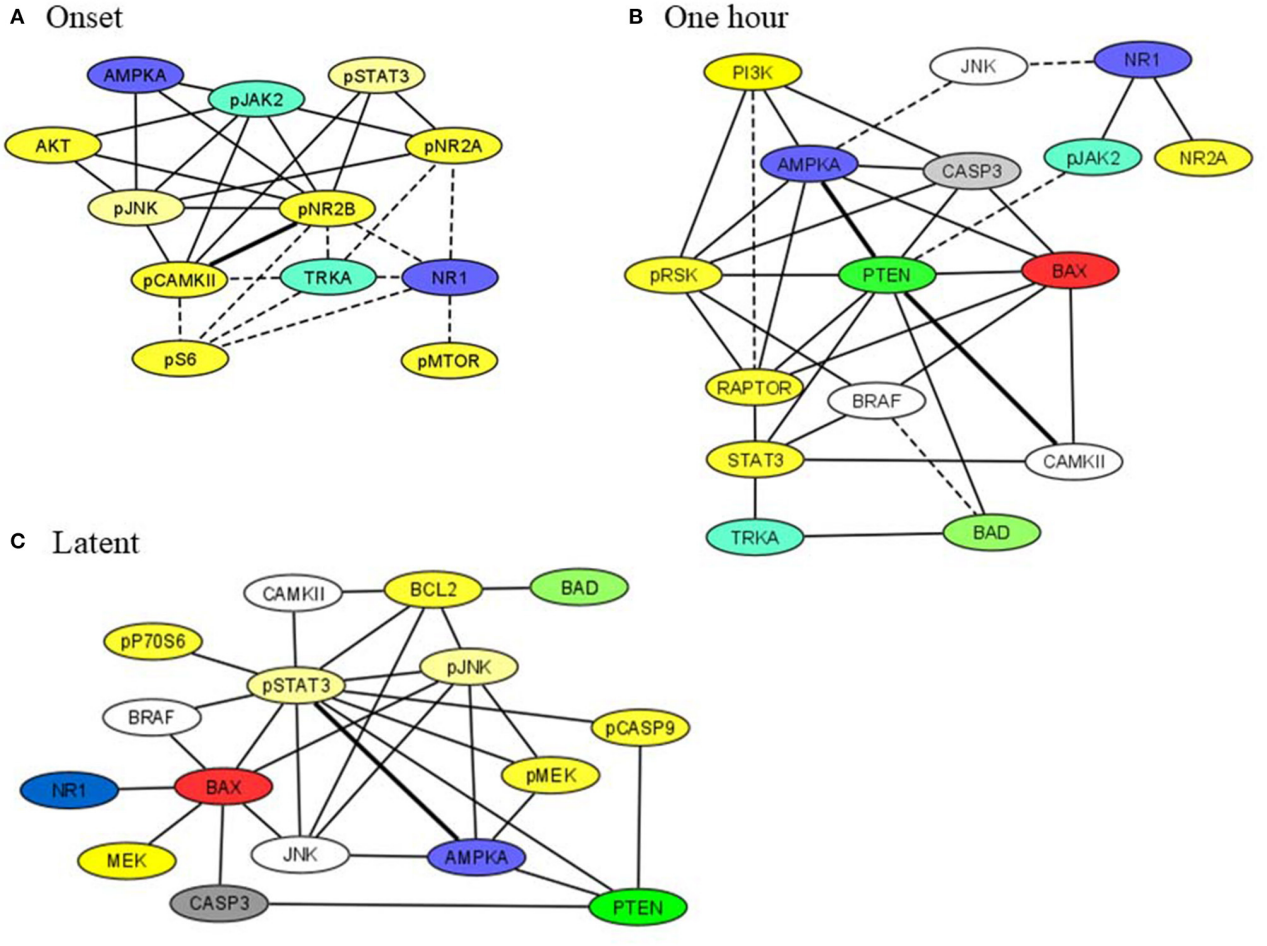
Correlation networks in hippocampus including STAT3, JAK2, and NMDAR subunits at **(A)** onset, **(B)** 1 h, **(C)** latent period. Correlations between protein levels were determined by Spearman Correlation analysis; only correlations with *r* > 0.8, with linear scatter plots and *p* < 0.05 were considered significant and are included. Heavy solid lines, correlations seen in pilocarpine and saline treated mice; thin solid lines, correlations seen only in pilocarpine treated mice; dashed lines, correlations seen only in saline treated mice. Colors indicate proteins present in two or three networks.

Subsets of proteins measured here interact as components of the same pathway, through crosstalk between pathways, as components of a complex or in modifier-substrate relationships. We therefore investigated possible correlations among levels of individual proteins in saline injected mice to compare their presence or loss in pilocarpine injected mice at corresponding time points (Spearman Correlation analysis). Within each brain region, scatter plots of all pairs of proteins in each treatment group with *r* > 0.8, *p* < 0.05 were manually reviewed and correlations with artifactual significance were eliminated. We then generated networks to illustrate selected relationships. Figure 4 shows networks involving STAT3, JAK2, and/or apoptosis related proteins, in hippocampus, at onset, 1 h and during the latent period. At onset (Figure 4A), in saline mice, levels of phosphorylated subunits if the NMDAR are correlated with each other and with NR1, TRKA, and phosphorylated MTOR pathway components, pS6 and pMTOR. These correlations are absent in pilocarpine mice, where the network instead uniquely includes NMDAR subunits correlated with pSTAT3, pJAK2, and AMPKA. Only a single correlation, between pCAMKII and pNR2B, is common to both treatment groups. By 1 h (Figure 4B), most of these relationships are lost. Phosphorylated NMDAR subunits, pSTAT3, and MTOR components are absent. In pilocarpine mice, PTEN now appears as a central hub connected with apoptosis proteins, BAD, BAX, and CASP3, among others that are mostly not present in the saline treated network. At the latent stage (Figure 4C), correlations remain predominant in pilocarpine mice. While PTEN remains in the network, now pSTAT3 is correlated with many proteins, directly with BAX, BCL2, pCASP9, and AMPKA, and indirectly with CASP3 and BAD. Saline mice have only a single correlation in common with pilocarpine mice, that between pSTAT3 and AMPKA.

**Figure 5:**
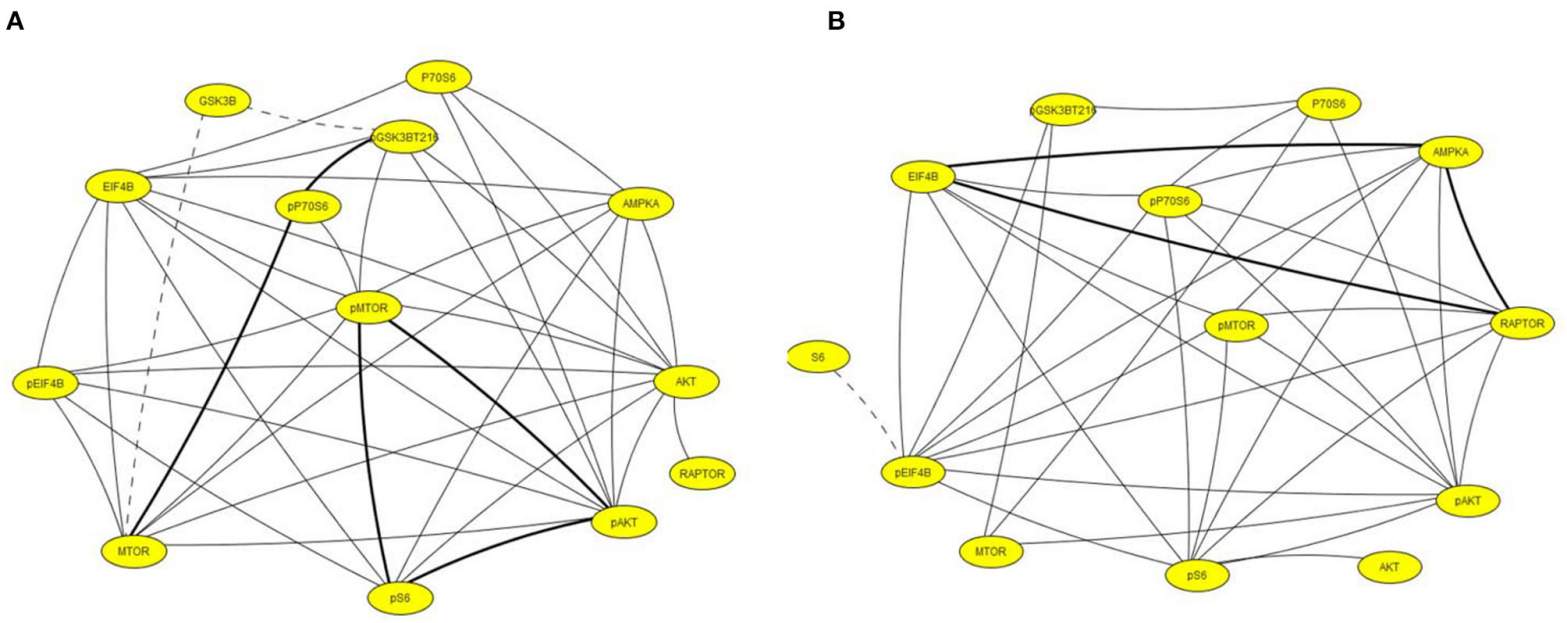
Correlation networks including components of the MTOR pathway in **(A)** hippocampus and **(B)** cortex. Correlations between protein levels were determined by Spearman Correlation analysis; only correlations with *r* > 0.8, with linear scatter plots and *p* < 0.05 were considered significant and are included. Heavy solid lines, correlations seen in pilocarpine and saline treated mice; thin solid lines, correlations seen only in pilocarpine treated mice; dashed lines, correlations seen only in saline treated mice.

Figure 5 illustrates networks of correlations at the chronic time point among components of the MTOR pathway in hippocampus and cortex. In contrast to the networks in Figure 4, correlations in saline mice are dominant. Levels of all components are highly correlated in both brain regions while in pilocarpine mice, there are only three correlations in common with saline mice in hippocampus and five in cortex.

## Discussion

The pilocarpine model of acquired TLE provides an opportunity to monitor molecular changes occurring throughout acquired epileptogenesis. Here, the technique of Reverse Phase Protein Arrays was used to assess responses to pilocarpine in signaling pathways and cellular processes known to underlie normal brain function and known or suspected to be involved in epileptogenesis. RPPA is a moderate throughput proteomics technique that is more conservative of sample and more sensitive than Western blots. For example, post-pilocarpine injection, three brain regions were assayed at six time points in 5 mice per time point. With saline injected mice, this resulted in a total of 180 samples, a number impractical for Western blots and mass spectrometric protocols. RPPA also allowed the assay of three replicates of a five point dilution series per sample, thus also providing more reliable measurements. The picture developed here shows complicated perturbations in multiple components of each pathway/cellular process assayed, and loss of normal correlations among functionally related proteins. Responses to pilocarpine are brain region-specific. In particular, while hippocampus and cortex are strongly affected, significant responses are also seen in cerebellum, a region recently receiving attention for contributions to epilepsy but not previously studied with respect to associated molecular processes. The current report represents an initial study of experiments carried out in only male animals. We recognize that these studies need to also be carried out in females to fully understand the pattern of gene expression associated with acquired epileptogenesis.

Many studies have analyzed rodent models of acquired epilepsy, using pilocarpine as here, or kainate, pentylenetrazole, or direct electrical stimulation. Variations in protocols include, not only the basic methodology, but also the number of time points measured, the brain region(s), and level of tissue resolution. A further complication is measurement of protein phosphorylation, a modification which is known to be labile and sensitive to alteration by both phosphatases and kinases that may be released from cellular sequestration immediately upon animal sacrifice and during tissue processing. Here mice were sacrificed by cervical dislocation without anesthetics, which are known to alter gene expression, and tissues were immediately heat stabilized to inactivate all enzymes involved in protein modification. Thus, results may differ from those in studies where anesthetics were used and/or tissue processing was prolonged due to perfusions. For all these factors, comparing results among studies that used different protocols is not necessarily straightforward.

Results for the MTOR pathway from several studies using western blots and either kainate or pilocarpine-induced SE are qualitatively consistent with results here. Increases in ratios of pMTOR/MTOR and pAKT/AKT in hippocampus from 2 to 16 h (29) and increases in pS6 in hippocampus and cortex at 1–6 h (12) after SE induced by kainate were replicated here, although a further peak in pS6 reported at 5 days was not observed here. Similar early increases in pS6 in hippocampus and cortex (30 min to 2 h) were also seen after pilocarpine-induced SE (30). MAPK components, pJNK, and pP38, have been reported to increase in the chronic stage after pilocarpine and pentylenetetrazole SE, respectively (28, 31); here, increases were seen in the sub-acute time points. The former study examined only the CA1 region of hippocampus, which clearly could differ in details that may be masked, by examining whole hippocampus.

Apoptosis related proteins function in neuronal cell death, and its prevention, after acute seizures and can activate a caspase cascade [reviewed in (32, 33)]. Detailed time frames of increases and decreases of BCL2, BAD, BAX, and CASP3 and CASP9 are determined here. SE and neuronal death have also been linked to STAT3 activation (34); here, STAT3 and pSTAT3 show complex patterns in both hippocampus and cortex throughout epileptogenesis, and STAT3 remains high during the chronic phase. These examples show that our results are consistent with prior data, supporting the validity of our datasets overall.

Large scale proteomics studies using 2D gels and/or mass spectrometry can identify hundreds to thousands of proteins and detect differential expression between treatment groups. Commonly, a few tens of differentially expressed proteins have been quantified (18, 19, 35, 36). The advantage of such approaches is that they are unbiased, thus, not limiting protein measurements to a specific hypothesis or pathway focus. None of these large-scale proteomics efforts has identified any of the proteins measured here. One possible reason is that mass spectrometry identifies more highly expressed proteins, unless extensive subcellular enrichment processes are used. Most such studies have applied bioinformatics techniques for search for pathway associations in their datasets. Of interest, this has identified enrichments in the MAPK pathway and inflammation processes.

This work for the first time examines perturbations in multiple pathways in the cerebellum during epileptogenesis. The cerebellum historically has received most attention for sensorimotor control, however increasingly data indicate a role for the cerebellum in cognition [reviewed in (37)]. Evidence for the cerebellum's involvement in epilepsy started with structural abnormalities (38), but now includes functional data from both human patients and rodent models [(39, 40); reviewed in (41)]. At the protein level, Rubio et al. (42) recently showed that levels of BAX and activated CASP9 increased and BCL2 decreased during chronic seizures induced by electrical stimulation. This is consistent with our observations regarding BAX and CASP9 (although not for BCL2). Our data demonstrate that common signaling pathways, MAPK and MTOR, are perturbed at onset of SE as well as through epileptogenesis, to extents similar to those seen in hippocampus and cortex.

The number and nature of the proteins measured allowed generation of correlation networks to provide a novel perspective of molecular events during epileptogenesis. While the functional implications, if any, remain to be determined, two features are notable. First, the hippocampus of pilocarpine treated mice shows uniquely complex networks involving JAK/STAT3 and apoptosis related proteins which evolve during epileptogenesis to contain PTEN. These robust correlations, that are almost entirely absent in saline mice, may contribute to or be reflective of the loss of normal regulation of synaptic activity and the development of highly synchronous neuronal firing underlying spontaneous seizures. Second, and conversely, during the chronic phase, components of the MTOR pathway are highly correlated in both hippocampus and cortex of saline mice. The almost complete absence of these networks in pilocarpine mice is consistent with the known role of mutations of MTOR pathway components in epilepsy. Additional networks can be constructed for hippocampus and cortex, as well as cerebellum; their brain region specificity supports their potential functional significance. Further studies are needed to ascertain the predictive value of these and other correlation networks.

Recent evidence suggests that the signaling pathways involving mTOR and MAPK activation are important regulators of synaptic excitability and might be responsible for epilepsy and the concomitant cognitive impairment (43). Cell-specific mutations of TSC genes induced in both glia and neurons cause epilepsy in mice (44–46). Further, the neuropathological hallmarks of TSC include major morphological and functional changes in glial cells involving astrocytes, oligodendrocytes, NG2 glia, and microglia, as well as in neurons (47), and abnormal function of the mTOR pathway in glial cells is thought to contribute to seizures as well as cognitive co-morbidities in TSC as well as in TLE (48, 49). Specimens surgically removed from people suffering from TLE show increased levels of pMTOR (S2448), pS6 (S235/236), and pS6 (S240/244) that are consistent with the activation of the mTOR pathway. More importantly, a correlation between the increase in the levels of pS6 (S235/236) and the localization of the seizure focus within the mesial temporal lobe structures can be detected (50). Loss of PTEN expression from granule cells of dentate gyrus promotes an increase in S6 phosphorylation associated with numerous cellular abnormalities including hypertrophy, basal dendrites, increased dendritic spine density and ectopically located somas (51). A correlation between mTOR hyperactivity and disease severity has been reported in a particular experimental model of focal malformations (52) produced by *in utero* electroporation of a constitutively active form of Rheb (Rheb^CA^), the canonical activator of mTORC1. In addition to promoting epilepsy and its associated pathologies, Rheb^CA^ overexpression promotes an increase in neuronal size and cell misplacement strongly suggesting that mTOR hyperactivation directly influences seizure severity (52). Excessive mTOR signaling leads to hippocampal hyperexcitability linking mTOR with TLE. Knockout of PTEN, promotes mTOR-signaling hyperactivation resulting in seizures that also mimic an epileptic phenotype (43). Thus, the relevance of mTOR in the control of excitation/inhibition is crucial for the homeostatic control of neuronal excitability.

The components of the MAPK signaling pathway include the extracellular signal-regulated kinases 1 and 2 (ERK1/2), c-Jun amino-terminal kinases 1-3 (JNK1/2/3), and p38^MAPK^ (a, b, d) (53). The MAPK signaling pathway is massively activated within hippocampus after acute administration of chemoconvulsants like kainate and pilocarpine (54) and was demonstrated here. However, pharmacological blockade of ERK has no effect on the initiation or severity of seizures supporting the notion that ERK activation is due to neuronal excitability rather than the actual cause of seizures (54). A previous study also demonstrated an increase in the phosphorylation of ERK during the acute period (1 and 12 h) post-pilocarpine-induced seizure in both the hippocampus and cortex, suggesting that ERK might be involved in epileptogenesis (55). These results were replicated here, continuing to 24 h. In addition, increased ERK phosphorylation has been reported in the temporal neocortex of patients with intractable epilepsy (56) and linked to persistent activation of MAPK-dependent gene transcription in brain regions with hyperconnected neurons of layer 2/3 (57). ERK also activates the expression of NMDA receptors leading to increased excitability and seizures. As a whole, these observations suggest that, since activation of the MAPK pathway occurs following many protocols for seizure, its activation is the result of seizure activity (43).

Activation of the JAK/STAT pathway has been demonstrated in the hippocampus in animal models of TLE, as well as in patients. Brain injuries leading to TLE (i.e., epileptogenic brain injuries) in rodents persistently alter JAK/STAT pathway activity (58–61). Both the phosphorylation and total expression of STAT3 is increased in tissue samples resected from patients with epilepsy due to hippocampal sclerosis (62), focal cortical dysplasia (62), and tumors (63). The JAK/STAT pathway is a critical cell signaling pathway in glia as well as neurons, and regulates gliogenesis (64), neuronal survival and maturation (65), and neuronal gene expression (66). The current study demonstrates that the activation of the JAK/STAT pathway that has previously been shown to occur acutely in hippocampus after epileptogenic brain insults, persists into the chronic epilepsy stage, and occurs outside of the hippocampus, in areas such as the cortex and cerebellum. Our findings provide further support for the concept that the JAK/STAT may play a pervasive and persistent role in the regulation of multiple mechanisms contributing to epileptogenesis.

In summary, the current findings expand our understanding of time- and brain region- specific changes in expression, activity, and correlations among levels of functionally related proteins affecting both neurons and glia. While hippocampus, the brain region most affected during pilocarpine-induced epileptogenesis, shows large numbers of perturbations, cortex and cerebellum also display significant and divergent responses in pathways that are critical for brain function. The results include demonstration of complex sequential alterations in levels of components of the MTOR, MAPK, JAK/STAT, and apoptosis pathways and in subunits of the N-methyl-D-aspartate receptor (NMDAR) receptor. We further demonstrate correlations among levels of pathway and receptor components in saline treated mice, that are lost over time and replaced with other patterns with pilocarpine.

Epilepsy is not a single disease but rather a constellation of disorders of different etiologies that share the common phenotypic feature of spontaneous seizures. In this manuscript we specifically focus on a preclinical rodent model of acquired epilepsy, with the intent of elucidating the array of molecular pathways that are activated during epileptogenesis and over what time-course. Understanding changes in pathway activation adds to the existent literature on altered gene expression, and provides new information about prospective targets for therapeutic intervention. The current data present a novel picture of the complexity of protein responses during epileptogenesis that may provide targets for novel therapeutics aimed at disrupting the epileptogenic processes and preventing or reducing development of spontaneous seizures. Future interventional studies that modify pathway activation will be required, however, to determine which, if any, of the protein responses here identified play a causal role in epileptogenesis and have potential as a target for disease-modifying therapy.

## Data Availability Statement

The original contributions presented in the study are included in the article/Supplementary Material, further inquiries can be directed to the corresponding author/s.

## Ethics Statement

The animal study was reviewed and approved by Institutional Animal Care and Use Committee of the University of Colorado Anschutz Medical Campus.

## Author Contributions

KG and AB-K conceived, designed and supervised the study, and obtained funding for the study. MA, AC, YC, JC, AT, and MG were involved in the execution of the experiments. MA, AC, and KG were involved in the data acquisition and interpretation. MA, AC, MG, KG, and AB-K were involved in the review and analysis of the data. MA, MG, KG, and AB-K participated in writing and editing the manuscript. All authors approved the final manuscript and are responsible for the work presented in the manuscript.

## Conflict of Interest

The authors declare that the research was conducted in the absence of any commercial or financial relationships that could be construed as a potential conflict of interest.

## Supplementary Material

Supplementary Figure 1 Chronology of the experiments. Male FVB/NJ mice were purchased from Jackson Labs and received at 5–7 weeks of age. Mice were allowed to acclimate to the environment for 1 week prior to the start of experiments. Mice (18–24 g) were briefly handled once daily for about 1 week to reduce the stress induced by experimental protocols. Entire litters (an average of 5 mice) were randomly assigned to a single treatment/time point group. Mice were given an intraperitoneal injection of scopolamine methyl bromide (1 mg/kg) 15 min before the first pilocarpine injection to block the peripheral effects of pilocarpine. The initial dose of pilocarpine HCl (200 mg/kg) was followed, after 1 h, by subsequent doses (100 mg/kg) given at 30 min intervals. The onset of SE was defined as the appearance of repeated behavioral seizures (stage four or higher with at least one seizure being five or higher) according to a modified Racine scale. SE persisted for at least 90 min. Control mice were given injections of saline at identical time intervals. After SE induction, mice were singly housed and given free access to water and moistened chow. Cohorts of 5 mice per group were sacrificed after SE, at 15 min (onset), 1 and 6 h, 1 and 5 days, and 2 weeks (red lines). Groups of saline-injected mice were sacrificed at the same time points.
