# Supplementary figures and images for: Altered Protein Profiles During Epileptogenesis in the Pilocarpine Mouse Model of Temporal Lobe Epilepsy
Source: Front Neurol. 2021 May 28;12:654606. doi: 10.3389/fneur.2021.654606 (PMC8194494; doi:10.3389/fneur.2021.654606)

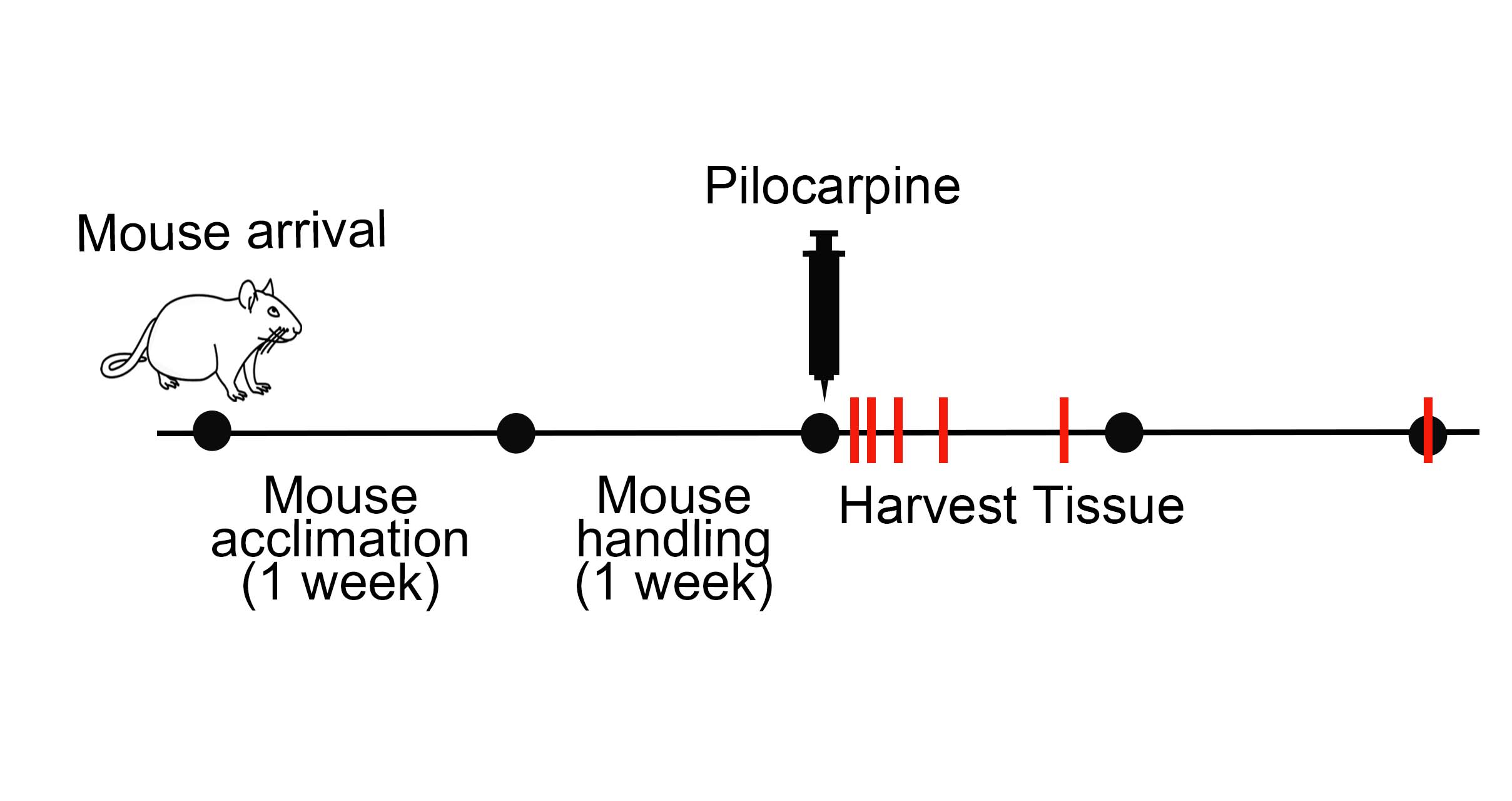

Supplement: Supplementary Figure 1 — Chronology of the experiments. Male FVB/NJ mice were purchased from Jackson Labs and received at 5–7 weeks of age. Mice were allowed to acclimate to the environment for 1 week prior to the start of experiments. Mice (18–24 g) were briefly handled once daily for about 1 week to reduce the stress induced by experimental protocols. Entire litters (an average of 5 mice) were randomly assigned to a single treatment/time point group. Mice were given an intraperitoneal injection of scopolamine methyl bromide (1 mg/kg) 15 min before the first pilocarpine injection to block the peripheral effects of pilocarpine. The initial dose of pilocarpine HCl (200 mg/kg) was followed, after 1 h, by subsequent doses (100 mg/kg) given at 30 min intervals. The onset of SE was defined as the appearance of repeated behavioral seizures (stage four or higher with at least one seizure being five or higher) according to a modified Racine scale. SE persisted for at least 90 min. Control mice were given injections of saline at identical time intervals. After SE induction, mice were singly housed and given free access to water and moistened chow. Cohorts of 5 mice per group were sacrificed after SE, at 15 min (onset), 1 and 6 h, 1 and 5 days, and 2 weeks (red lines). Groups of saline-injected mice were sacrificed at the same time points. [file Image_1.jpeg]
