# Supplementary material for: Altered Protein Profiles During Epileptogenesis in the Pilocarpine Mouse Model of Temporal Lobe Epilepsy
Source: Front Neurol. 2021 May 28;12:654606. doi: 10.3389/fneur.2021.654606 (PMC8194494; doi:10.3389/fneur.2021.654606)
Supplement: Supplementary file 2 [file Table_1.DOC]

**Supplementary Table S1. Protein and antibody information.** All proteins used in RPPA are included, with common and official names. Functional class: ID, mutation in the human gene results in intellectual disability (Sturgeon et al 2012). LM, SP and ST: mutation of the mouse gene results in abnormal learning/memory, synaptic plasticity and synaptic transmission, respectively (information from the Mammalian Phenotype Browser, <http://www.informatics.jax.org/searches/MP_form.shtml>); Y, yes. Other: MAPK, protein is a component mitogen activated protein kinase pathway; MTOR: protein is a component of the mechanistic target of rapamycin pathway; apoptosis, protein functions in apoptosis; IEG, immediate early gene; AD, reported abnormal in brains of patients with Alzheimer’s Disease or mouse models of AD

| **Common protein name** | **Official name** | **Functional class** | | | | | | | | **Antibody Source** | **Catalogue #** | **Dilution** |
| --- | --- | --- | --- | --- | --- | --- | --- | --- | --- | --- | --- | --- |
|  |  | **ID** | **LM** | | **SP** | | **ST** | **Other** | |  |  |  |
| AKT | AKT1 |  |  | |  | | Y | MTOR | | Santa Cruz Biotechnology | SC-1619 | 1:300 |
| AMPKA | PRKAA1 |  |  | |  | |  | MTOR | | Cell Signaling | 2532 | 1:750 |
| BAD | BAD |  |  | |  | |  | Apoptosis | | Cell Signaling | 9292 | 1:500 |
| BAX | BAX |  |  | |  | |  | Apoptosis | | Cell Signaling | 2772 | 1:750 |
| BCL2 | BCL2 |  |  | |  | |  | Apoptosis | | Cell Signaling | 2870 | 1:500 |
| BRAF | BRAF | Y | Y | |  | | Y | MAPK | | Cell Signaling | 9434 | 1:500 |
| CAMKII | CAMK2 |  | Y | | Y | | Y | MAPK | | Cell Signaling | 3362 | 1:500 |
| CASP3 | CASP3 |  | Y | | Y | | Y | Apoptosis; AD | | Cell Signaling | 9662 | 1:500 |
| CASP9 | CSP9 |  | Y | | Y | | Y | Apoptosis;AD | | Novus Biologicals | NB100-56366SS | 1:500 |
| CDK5 | CDK5 |  | Y | |  | | Y | AD | | Cell Signaling | 2506 | 1:500 |
| CREB | CREB1 |  | Y | |  | | Y | MAPK | | Cell Signaling | 9197 | 1:500 |
| EIF4B |  |  |  | |  | |  |  | | Novus Biologicals | NBP2-24504SS | 1:500 |
| ERK1/2 | MAPK1/3 |  | Y | |  | | Y | MAPK | | Santa Cruz Biotechnology | SC-153 | 1:300 |
| GluR3 | GRIA3 | Y |  | |  | | Y | iGlut | | Cell Signaling | 5117 | 1:750 |
| GluR4 | GRIA4 |  | Y | |  | | Y | iGlut | | Cell Signaling | 3824 | 1:300 |
| GSK3B | GSK3B |  | Y | |  | | Y | MTOR; AD | | BD Biosciences | 610201 | 1:3000 |
| JNK | MAPK8 |  |  | |  | |  | MAPK | | Cell Signaling | 9252 | 1:500 |
| MEK1/2 | MAP2K1/2 | Y |  | |  | |  | MAPK | | Cell Signaling | 9122 | 1:500 |
| MTOR | MTOR |  |  | |  | |  | MTOR | | Cell Signaling | 2972 | 1:500 |
| NR1 | GRIN1 | Y | Y | | Y | | Y | AD; iGlut | | Upstate Biotechnology | 07-362 | 1:500 |
| NR2A | GRIN2A | Y | Y | |  | | Y | AD; iGlut | | PhosphoSolutions | 1497-NR2A | 1:500 |
| P35/25 | CDK5R1 |  | Y | |  | | Y | AD | | Cell Signaling | 2680 | 1:750 |
| P38 | MAPK14 |  |  | |  | |  | MAPK | | Cell Signaling | 9212 | 1:500 |
| P70S6 | RPS6KB1 |  |  | |  | |  | MTOR | | Santa Cruz Biotechnology | SC-8418 | 1:400 |
| pAKT (Ser473) | AKT1 |  |  | |  | | Y | MTOR | | Cell Signaling | 4060 | 1:500 |
| pBRAF(Thr401) | BRAF | Y | Y | |  | | Y | MAPK | | Epitomics | 2298-1 | 1:1000 |
| pCAMKIIA/B(Thr286) | CAMK2A/B |  | Y | | Y | | Y | MAPK | | PhosphoSolutions | p1005-286 | 1:4000 |
| pCASP9(Ser196) | CAPS9 |  |  | |  | |  | Apoptosis | | Abgent | AP3044a | 1:300 |
| pCREB( Ser133) | CREB1 |  | Y | |  | | Y | MAPK | | PhosphoSolutions | p1010-133 | 1:500 |
| pEIF4B(Ser422) | EIF4B |  | |  | |  |  | | MTOR | Cell Signaling | 3591 | 1:500 |
| pELK1 (Ser383) | ELK1 |  |  | |  | |  | MAPK | | Santa Cruz Biotechnology | SC-8406 | 1:500 |
| pERK1/2(Tyr204) | MAPK1/3 |  | Y | |  | | Y | MAPK | | Santa Cruz Biotechnology | SC-7383 | 1:500 |
| pGluR2(Tyr876) | GRIA2 |  | Y | | Y | | Y | iGlut | | Cell Signaling | 4027 | 1:500 |
| pGSK3β (Tyr216) | GSK3B |  | Y | |  | | Y | MTOR; AD | | BD Biosciences | 612312 | 1:2000 |
| P13K |  |  |  | |  | |  |  | | Cell Signaling | 4249 | 1:500 |
| pJAK2 (Tyr1007/1008) | JAK2 |  |  | |  | |  |  | | Santa Cruz Biotechnology | SC-21870 | 1:200 |
| pJNK(Thr183/Tyr185) | MAPK8 |  |  | |  | |  | MAPK | | Cell Signaling | 9251 | 1:1000 |
| pMEK1/2(Ser217/221) | MAP2K1/2 | Y |  | |  | |  | MAPK | | Cell Signaling | 9154 | 1:750 |
| pMTOR(Ser2448) | MTOR |  |  | |  | |  | MTOR | | Cell Signaling | 2971 | 1:500 |
| pNR1(Ser889) | GRIN1 | Y | Y | | Y | | Y | AD; iGlut | | Epitomics | 2329-1 | 1:500 |
| pNR2A(Tyr1246) | GRIN2A | Y | Y | | Y | | Y | AD;iGlut | | Cell Signaling | 4206 | 1:500 |
| pNR2B(Tyr1336) | GRIN2B | Y | Y | | Y | | Y | AD; iGlut | | PhosphoSolutions | p1516-1336 | 1:500 |
| pP38 |  |  |  | |  | |  |  | |  |  |  |
| pP70S6(Thr389) | RPS6KB1 |  |  | |  | |  | MTOR | | Cell Signaling | 9205 | 1:500 |
| pPKCA/B(Thr638/641) | PRKAA/B |  |  | |  | |  | MAPK | | Cell Signaling | 9375 | 1:750 |
| pRSK(Ser380) | RPS6KA3 | Y |  | |  | |  | MAPK | | Cell Signaling | 9341 | 1:500 |
| pS6(Ser240/244) | RPS6 |  |  | |  | |  | MTOR | | Cell Signaling | 5364 | 1:500 |
| pSTAT3 |  |  |  | |  | |  |  | |  |  |  |
| PTEN |  |  |  | |  | |  |  | | Cell Signaling | 5384 | 1:500 |
| RAPTOR | RPTOR |  |  | |  | |  | MTOR | | Cell Signaling | 2280 | 1:750 |
| RSK2 | RPS6KA3 | Y |  | |  | |  | MAPK | | Cell Signaling | 9340 | 1:300 |
| S6 | RPS6 |  |  | |  | |  | MTOR | | Cell Signaling | 2217 | 1:500 |
| STAT3 | STAT3 |  |  | |  | |  | JAK-STAT | | Cell Signaling | 9139 | 1:500 |
| TRKA | NTRK1 | Y | Y | |  | |  | AD | | Epitomics | 2244-1 | 1:2000 |
